# Supplementary material for: WordCommentsAnalyzer: A windows software tool for qualitative research
Source: F1000Res. 2018 Sep 4;7:536. Originally published 2018 May 3. [Version 2] doi: 10.12688/f1000research.14819.2 (PMC6137411; doi:10.12688/f1000research.14819.2)
Supplement: Supplementary file 3 [file f1000research-7-17614-s0002.tgz › b2a79496-4ada-47c4-9682-a99ce867836d.zip!/Eight Iranian Health Journals/Journal of Research in Rehabilitation Sciences _23.docx]

© Journal of Research in Rehabilitation Sciences . This work is licensed under CC BY-NC 4.0 (https://creativecommons.org/licenses/by-nc/4.0/).

Volume & Issue: دوره 10، شماره 2: 1393

Title: مقایسه دامنه حرکتی و استقامت عضلانی ناحیه گردن در بیماران مبتلا به سردرد تنشی با افراد سالم

http://jrrs.mui.ac.ir/index.php/jrrs/article/view/1093

چکیده   مقدمه: سردرد یکی ازعلل اصلی شکایت بیماران است. 47% سردردها از نوع تنشی می باشند. سردردهای تنشی مرتبط با وضعیت عضلات اسکلتی می باشند و سفتی عضلات اطراف سر و گردن می تواند باعث بروز آنها شود. هدف از این مطالعه، مقایسه دامنه حرکتی فعال و استقامت عضلات فلکسور و اکستانسور گردن در افراد مبتلا به سردرد تنشی و افراد سالم می باشد.   مواد و روش ها: جامعه مورد پژوهش شامل52 دختر سالم و 52 دختر مبتلا به سردرد تنشی با محدوده سنی 20 تا 25 سال است. میزان دامنه حرکتی اکتیو در دو جهت فلکسیون و اکستانسیون و استقامت عضلات فلکسور و اکستانسور گردن در این نمونه ها، تعیین گردید.   یافته ها: هیچ اختلاف معناداری بین دامنه حرکتی اکتیو و استقامت عضلانی ناحیه گردن در دو گروه مبتلا به سردرد تنشی و گروه سالم مشاهده نشد (p>0.05).   نتیجه گیری: افراد مبتلا به سردرد تنشی در دامنه سنی 20 تا 25 سال از نظر استقامت عضلات اطراف گردن و دامنه حرکتی گردن تفاوت چندانی با افراد سالم ندارند و احتمالا در افراد مبتلا به سردرد تنشی با گذشت زمان تغییرات اسکلتی-عضلانی در ستون فقرات و عضلات گردنی ایجاد می شود.

Title: بررسی ارتباط بین پرخیشومی شدگی گفتار با نوع شکاف و سن جراحی اولیه ی کام در کودکان 3 تا 6 سال دچار شکاف کام شهر اصفهان

http://jrrs.mui.ac.ir/index.php/jrrs/article/view/1204

مقدمه:   انواع مختلفی از شکاف کام اولیه و ثانویه با گستره های گوناگون وجود دارد. برخلاف تاریخچه ی نسبتاً طولانی جراحی کام، اتفاق نظر کمی در خصوص بهترین تکنیک های جراحی و حتی توافق نظر کم تری در خصوص بهترین زمان جراحی وجود دارد. در حال حاضر هیچ پروتکل استانداردی وجود ندارد تا معضل زمانبرای ترمیم شکاف کام را برای دستیابی به گفتار بهینه برطرف نماید. این مطالعه جهت اندازه گیری تعداد کودکان پیش دبستانی که هایپرنیزالیتی نشان می دادند، انجام شد. تأثیر جراحی اولیه ی کام و نوع شکاف روی هایپرنیزالیتی متوسط و شدید هم بررسی شد.   مواد و روش ها:   یک گروه از 46 کودک پیش دبستانی 3 تا 6 ساله که جراحی شکاف کام را انجام داده بودند، به وسیله ی سیستم پارامترهای جهانی برای گزارش برون دادهای گفتاری در کودکان با شکاف کام- نسخه ی فارسی، ارزیابی شدند.در انواع شکاف ها، شکاف دوطرفه ی لب و کام(BCLP; n=5,10/9%) شکاف یک طرفه ی لب و کام(UCLP; n=20,43/6%) ، شکاف سخت کام و نرم کام(CP;n= 10, 21/71%) ، شکاف نرمکام تنها(SPO;n=6, 13%) و شکاف کام زیر مخاطی(SUB.M.C;n=5, 10/9%)، قضاوت هایپرنیزالیتی توسط یک آسیب شناس گفتار و زبان باتجربه براساس مقیاسی 4 درجه ای انجام شد. 32 نفر جراحی کام را قبل از 12 ماهگی داشتند، 8 نفر بین 18-12 ماهگی جراحی کام را دریافت کرده بودند و 6 نفر بعد از 18 ماهگی جراحی شده بودند.   یافته ها :   %3/78از کودکان هایپرنیزالیتی متوسط تا شدید نشان دادند، که هیچ رابطه ی معناداری بین 5 گروه شکاف وجود نداشت. تجزیه و تحلیل ها، همچنین نشان دادند که هیچ رابطه ی معناداری بین جراحی اولیه ی کام و تعداد کودکانی که هایپرنیزالیتی قابل توجهی داشتند، وجود نداشت.   نتیجه گیری:   از آن جا که، شدت هایپرنیزالیتی در تمام کودکان بالا است، نیاز به تحقیقات مستند در زمینه ی میزان اثربخشی جراحی صورت گرفته روی این کودکان ضروری است. در این مطالعه گستره ی سنی وسیع بود و تعداد کودکان در هر گروه شکاف محدود بود. مطالعات بیش تری نیاز است تا تعیین کنند که آیا متغیرهای دیگری نیز وجود دارند که ممکن است تأثیر جراحی اولیه و نوع شکاف را مخفی کنند.

Title: آناليز فركانس‌هاي بحراني اندام‌هاي بدن راننده تراكتور

http://jrrs.mui.ac.ir/index.php/jrrs/article/view/1254

چكيده مقدمه: از ويژگي‌هاي سيستم‌هاي ارتعاشي، فركانس‌هاي بحراني آن است كه نقش بسزايي در ميزان ارتعاشات انتقال يافته به آن دارد. وقتي فركانس تحريك وسيله‌اي، در محدوده فركانسي اندام‌هاي بدن راننده باشد، مقدار ارتعاشات وارد بر بدن راننده افزايش يافته و باعث بروز آسيب‌هايي به بدن او مي‌گردد. اين در حاليست كه بكارگيري ارتعاشات در دامنه و فركانس‌هاي مناسب، باعث افزايش قدرت ديناميكي عضلات، بهبود دردهاي آن‌هاو كمردرد‌هاي مزمن، درمان پوكي استخوان و‌ انعطاف پذيري عضلات شود. لذا هدف از اين پژوهش تعيين فركانس‌هاي بحراني اندام‌هاي بدن راننده است. مواد وروش‌ها: جهت تعيين فركانس‌هاي بحراني راننده تراكتور در راستا‌هاي مختلف طولي، جانبي و عمودي، سه راننده با جرم‌هاي 55، 70، 85 كيلوگرم در اين آزمايش‌ها شركت نمودند.آزمايش‌ها در چهار شتاب تحريك 5/0، 1، 5/1 و 2 متر بر مجذور ثانيه و فركانس‌هاي مختلف انجام شد و مقادير شتاب‌هاي انتقال يافته به اندام‌هاي بدن راننده‌ها اندازه‌گيري شد.سپس فركانس‌هاي بحراني اندام‌هاي بدن راننده در راستاي‌هايمختلف تعيين گرديد. همچنين توسط آناليز رگرسيون چند متغيره‌، مقادير فركانس‌هاي بحراني اندام‌ها برآورد و محاسبه شدند. نتايج: نتايج بيانگر افزايش مقدار ارتعاش طولي در فركانس‌هاي 3-4 هرتز براي اندام‌هاي بدن بودند. در راستاي عمودي دو مد فركانسي ظاهر شد كه فركانس مد اول برابر 17-13 هرتز و فركانس مد دوم 11-9 هرتز بدست آمد. در راستاي جانبي نيز دو مد فركانسي 17-14 هرتز و 5/4 – 5/2 هرتز بدست آمد. بحث:با توجه به فركانس‌هاي بحراني بدست آمده در اين پژوهش، اعمال ارتعاشات به منظور افزايش پارامترهاي عملكردي عضلات اندام‌هاي مختلف بايستي در فركانس‌هاي بيش از 20 هرتز صورت پذيرد تا به بافت‌هاي بدن آسيب نرساند. همچنين روش محاسبه فركانس‌هاي طبيعي اندام‌هاي بدن راننده از طريق جرم راننده و فركانس تحريك، بيشترين تطابق را با نتايج حاصل از آزمايش‌هاي ايستگاهي داشت.

Title: تاثیر تمرینات منتخب یوگا بر تعادل پای غالب و غیر غالب زنان میانسال

http://jrrs.mui.ac.ir/index.php/jrrs/article/view/1383

چکیده مقدمه: تعادل یکی از مفاهیم مهم در دوران سالمندی است. بهبود تعادل در دوران میانسالی میتواند از سقوط سالمندان که هزینه های روانی و مالی بسیاری را به جامعه تحمیل می کند، پیشگیری کند. عدم توازن بین تعادل پای غالب و غیر غالب بدن نیز می توانند منجر به بروز برخی مشکلات شود. بنابراین بررسی میزان تاثیر تمرینات مشابه بر عضو غالب و غیر غالب بدن می تواند از بروز هر گونه مشکل ناشی از عدم توازن بین اعضای بدن جلوگیری کند. مواد و روش ها: 22 زن میانسال (میانگین سنی 89/4±18/54)، بدون هیچگونه سابقه بیماری در این تحقیق شرکت کردند. در جلسه اول تعادل پویا با استفاده از آزمون ستاره و تعادل ایستا توسط ایستادن بر روی یک پا در پای غالب و غیر غالب اندازه گیری شد. شرکت کنندگان 24 جلسه به اجرای تمرینات منتخب یوگا پرداختند. در جلسه آخر تعادل پویا و ایستا مجددا اندازه گیری شد. یافته ها: نتایج آزمون تی نشان داد که تعادل ایستا و پویا در پای غالب و غیر غالب به طور معناداری پس از 8 هفته تمرین یوگا افزایش یافت (p<0.05). همچنین بین میزان بهبود پای غالب و غیر غالب در تعادل پویا تفاوت معناداری یافت شد (p<0.05). اما تعادل ایستای هر دو پا به یک اندازه بهبود یافته بود. نتیجه گیری: می توان به این نتیجه رسید که تمرینات یوگا منجر به افزایش تعادل ایستا و پویا در هر دو پا می شود. تعادل پویای پای غیر غالب به طور معناداری بیشتر از پای غالب بهبود یافته بود اما تعادل ایستای پای برتر و غیر برتر به یک میزان افزایش یافت. بنابراین به نظر می رسد که در درمان های کلینیکی و تمرینات ورزشی تفاوت های موجود در تاثیرپذیری پای غالب و غیر غالب از تمرین باید به دقت مورد توجه قرار گیرد.

Title: انطباق پرسشنامه ی تجدید نظر شده برقراری ارتباط-نسخه دومChildren’s Communication Checklist (CCC-2 ) با زبان فارسی و تعیین ویژگی های روان سنجی آن

http://jrrs.mui.ac.ir/index.php/jrrs/article/view/1662

چکیده مقدمه:بیشتر آزمون­ها در گفتاردرمانی مبتنی بر ساختار زبان هستند و بنابراین برای ارزیابی کاربرد زبان نیاز به تهیه­ی ابزارهایی روا و معتبر می­باشد. پرسشنامه­ی برقراری ارتباط (1998) و نسخه­ی تجدید نظر شده­ی آن(2003) به ارزیابی کاربرد زبان اختصاص دارد. این مطالعه، با هدفترجمه انطباق و بومی­سازی پرسشنامه­ی  تجدید نظرشده برقراری ارتباط به زبان فارسی از طریق تعیین روایی محتوایی و بررسی  اعتبار آن(اعتبار درونی، اعتبارآزمون باز آزمون)انجام شده است. مواد و روش­ها:مطالعه حاضریک مطالعه غیر تجربی از نوع ابزارسازی بود. ترجمه پرسشنامه و برگردان ترجمه،  توسط سه آسیب­شناس گفتار و زبان و زبان­شناس صورت گرفت. همین­طور از نظرات 28 والد کودک 7تا 9 ساله دارای رشد طبیعی زبان در خصوص قابل درک بودن و ساده بودن گویه­ها استفاده شد. فرم ترجمه­شده پرسشنامه جهت تعیین روایی محتوایی برا­ی 10 آسیب شناس گفتار و زبان و زبان شناس فرستاده شد. سپس شاخص روایی محتوا(Content validity index)محاسبه گردید.ثبات درونی ابزار و اعتبار آزمون باز آزمون از طریق تکمیل پرسشنامه توسط98 والد کودک 7تا 9 ساله طبیعی صورت گرفت. یافته­ها:در بخش ترجمه و انطباق به جز 4 گویه، سایر گویه­هاCVRبالاتر از 0.85 احراز کردند که پس از اصلاح مجدد، این گویه­هانیز امتیاز مناسب کسب کردند. شاخص روایی محتوایی گویه­ها (CVI) بالاتر از 0.75 بود.ضریب آلفای کرونباخ زیرمجموعه­ها بین 0.66 تا 0.74 محاسبه شد و نتایج بازآزمایی  بعد از دوهفته بالاتر از 0.90 بدست آمد. نتیجه­گیری: بر طبق نتایج حاصل از پژوهش حاضر به نظر می­رسدکه پرسشنامه مطابقت داده شده­ی برقراری ارتباط کودکان به زبان فارسی از رواییمحتوا  و پایایی قابل قبولی برای کودکان 7 تا 9 سالهبرخوردار است. با این وجود نیاز به مطالعات دیگری به منظور سنجش بهتر ویژگی­های روان­سنجی این پرسشنامه در نمونه هایی با حجم بالاتر و در گروه­های سنی دیگر به چشم می خورد.

Title: بررسی اثربخشی تمرینات اصلاحی بر مولفه عمودی نیروی عکس العمل زمین در راه رفتن دانش آموزان پسر مبتلا به صافی کف پا

http://jrrs.mui.ac.ir/index.php/jrrs/article/view/1419

چکیده : مقدمه: در بعضی افراد به دلیل عدم کارکرد مناسب عضلات پا، قوس کف پایی دچار افت شده و به نظر مي رسد که نيروي عکس العمل زمین این افراد نیز نسبت به افراد داراي پاي نرمال، به علت کاهش جذب نیرو توسط این قوس دچار تغییر شود. هدف پژوهش حاضر بررسی اثر بخشی تمرینات اصلاحی بر مولفه عمودی نیروی عکس العمل زمین در حین راه رفتن کودکان مبتلا به صافی کف پا بود. مواد و روش ها: در این تحقیق 30 نفر دارای ناهنجاری کف پای صاف به شکل تصادفی و همگن در دو گروه مداخله و کنترل تقسیم شدند. افراد گروه مداخله 24 جلسه به مدت دو ماه هر هفته سه جلسه در تمربنات اصلاحی شرکت کردند. برای اندازه گیری نیروی عکس العمل زمین از دستگاه صفحه نیروی سنج(force plate)  دقیقا قبل و بعد از تمرینات اصلاحی استفاده گردید. جهت تجزیه و تحلیل آماری از آزمون هاي پارامتري t همبسته و t مستقل(  independent t-test و paired sample t-test) استفاده شد( 05/0≥ p). نتایج : نتایج این تحقیق نشان داد که مولفه عمودي نيروي عکس العمل زمين در زمان انتقال وزن بر روي پاشنه(01/0=p) و پنجه (03/0=p) در مرحله Stance در گروه مداخله بعد از 8 هفته تمرين اصلاحي به طور معني داري کاهش پيدا کرده است. ولي درزمان تماس کف پا با زمين، اختلاف معني داري بين دو گروه مشاهده نشد(05/0<p).  بحث: بر اساس یافت های این تحقيق می توان نتیجه گرفت، حرکات اصلاحی می تواند کارایی قوس های کف پایی را جهت جذب نیروهای وارده افزایش و نیروی عکس العمل زمین افراد دارای کف پای صاف را در دو مرحله انتقال وزن بر روی پاشنه و انتقال وزن بر روی پنجه کاهش دهد.

Title: بررسي همبستگي بين آزمون هاي کفایت حرکتی بروینینکز ازرتسکی و مقیاس رشدی - حرکتی پی بادی درارزيابي مهارتهاي حركتي كودكان آموزش پذير مبتلا به ناتواني هوشي

http://jrrs.mui.ac.ir/index.php/jrrs/article/view/1307

چکیده : مقدمه:کودکان با ناتوانی هوشی و رشدی در عملکردهای حرکتی محدودیت ها و نقص هایی دارند . بنابراین شناخت و ارزیابی مشکلات حرکتی این کودکان در سنین پیش از دبستان جهت ارائه خدمات درمانی مناسب ضروری می باشد. هدف این پژوهش، تعیین همبستگی بین آزمونهایمهارت حرکتی بروینینکز ازرتسکی و مقیاس رشد حرکتی پی بادی -2 درکودکان با ناتوانی هوشی و رشدی آموزش پذیر بود . مواد و روش ها:در این مطالعه مهارتهای حرکتی (درشت ، ظریف)  60 کودک باناتوانی هوشی و رشدی 83-54 ماهه به وسیله تست های  BOTMP وPDMS2مورد ارزیابی قرار گرفت . یافته ها :نتایج نشان داد که بیندو تست حرکتی در نمرات کل (91/0 – 65/0 =r )، نمرات حرکات درشت              (88/0-58/0 r=) و نمرات حرکات ظریف (88/0-60/0 r=) همبستگی متوسط تا بالایی وجود داشته است . نتیجه گیری: با توجه به همبستگی متوسط تا بالای بین دو تست   BOTMP و PDMS2 درمانگر می تواند جهت ارزیابی مهارتهای حرکتی کودکان با ناتوانی هوشی و رشدی از یکی از اینآزمون ها استفاده کند و با توجه به آیتم های مورد ارزیابی و زمان آزمون ها به نظر می رسد در این گروه از کودکان آزمونPDMS2  بهتر از آزمونBOTMP بوده  است .

Title: شبیه سازی پاسخ فیبرهای عصبی در پاسخ به تحریک با جریان های تداخلی

http://jrrs.mui.ac.ir/index.php/jrrs/article/view/1506

چکیده مقدمه: جریان­ تداخلی یکی از محبوب­ترین جریان­های مورد استفاده در الکتروتراپی است. علی­رغم این محبوبیت، مطالعات اندکی به بررسی نحوه­ی متأثر شدن فیبرهای عصبی از این نوع تحریک پرداخته­اند. هدف این مطالعه توسعه مدلی نزدیک به واقع برای شبیه­سازی اثر جریان تداخلی بر فیبرهای عصبی و استفاده از آن جهت بررسی الگوی مکانی و زمانی فعالیت آن­ها در پاسخ به این جریان بوده است. اثر فرکانس مدولاسیون بر فعالیت عصبی حاصل نیز بررسی شده است. مواد و روش­ها: در این  مطالعه مدلی از بافت بدن شامل لایه­های پوست، چربی و ماهیچه در نرم­افزار COMSOL پیاده­سازی شد تا توزیع پتانسیل حاصل از اعمال جریان­ بدست آید. سپس از مدل دقیق MRG برای فیبرهای حسی و حرکتی استفاده شد و پاسخ آن­ها در محل­های مختلفی از مدل برای فرکانس حامل 4 کیلوهرتز، مدولاسیون 50 تا 250 هرتز و دامنه 1 تا 99 میلی­آمپر بررسی شد. یافته­ها: در پاسخ به تحریک، فیبرهای حسی که از زیر الکترودها گذر می­کنند، مسدود می­شوند ولی فیبرهای حسی و حرکتی در محل­های دیگر از بافت فعال می­شوند. الگوی زمانی فعالیت فیبرها وابسته به دامنه­ی تحریک و فرکانس مدولاسیون یا به صورت یک الگوی متناوب شامل دوره­های آتش - سکوت و یا به صورت آتش پیوسته با فرکانسی برابر با فرکانس مدولاسیون یا دو برابر آن است. هرچند در یک عمق مشخص آستانه­ی تحریک در مکان­های مختلف نسبت به الکترودها متفاوت است، اما فیبرها حتی در خارج از ناحیه­ی محصور بین الکترودها نیز فعال می­شوند. نتیجه­گیری: نتایج این مطالعه توجیه کننده­ی کاهش آزردگی ناشی از اعمال جریان تداخلی در محل الکترودها است و احتمال اثرگذاری جریان بر بافت­های غیر هدف را به هنگام تحریک تداخلی نشان می­دهد. همچنین تأیید کننده­ی ادعاهای سنتی مبنی بر نقش فرکانس مدولاسیون در تعیین فرکانس آتش شدن فیبر عصبی است.

Title: بررسی پایایی آزمون ـ بازآزمون و بین آزمونگران تست عملکرد دست جبسون تیلور در کودکان فلج مغزی

http://jrrs.mui.ac.ir/index.php/jrrs/article/view/1089

چکیده مقدمه: مشکلات عملکردی اندام فوقانی از رایج ترین اختلالاتی است که به دنبال فلج مغزی بوجود می آیند. کودکانی که بعلت مشکلات عصبی ـ عضلانی مانند فلج مغزی در عملکرد دست دچار مشکل هستند، فرصت کمتری برای دریافت اطلاعات از محیط و درک تاثیر عملکردشان بر محیط دارند و در نتیجه تجربه ی آنها در انجام فعالیتها کاهش می یابد. با توجه به میزان اختلال عملکرد دست و اهمیت آن در کودکان فلج مغزی بر آن شدیم که پایایی تست جبسون تیلور را در کودکان فلج مغزی مورد بررسی قرار دهیم. مواد و روش ها: پژوهش از نوع غیر تجربی و روش شناختی و به صورت تحلیلی و توصیفی است. در این مطالعه 33 کودک فلج مغزی دایپلژی 12- 8 ساله به صورت نمونه گیری غیراحتمالی ساده بررسی شد که این کودکان از هردو جنس بودند و از تمام مدارس استثنایی جسمی حرکتی سطح تهران انتخاب شدند. در این مطالعه عملکرد دست با حضور همزمان دو کارشناس بوسیله تست جبسون تیلور ارزیابی شد( پایایی بین آزمونگر). سپس یک هفته بعد، یک آزمونگر ارزیابی را دوباره انجام داد(پایایی درون آزمونگر) و با استفاده از ضریب همبستگی درونی، پایایی بررسی شد. یافته ها: نتایج نشان دهنده همبستگی بالا در توافق دو آزمونگر (P=0/001 و ICC=0/998) و توافق در یک آزمونگر (P=0/001 و ICC=0/95) بوده است. نتیجه گیری: نتایج تحقیق نشان داد که در کودکان دایپلژی 12- 8 ساله پایایی آزمون ـ بازآزمون و بین آزمونگران تست جبسون تیلور بالا بوده است. بنابراین به عنوان یک ابزار کلینیکی پایا برای ارزیابی عملکرد دست این کودکان پیشنهاد می شود.

Title: ترجمه و بررسی روایی وپایایی پرسشنامه مقیاس فردی لکنت

http://jrrs.mui.ac.ir/index.php/jrrs/article/view/1442

چکیده:   زمینه و هدف: مقیاس­های­ ­ فردی ابزارهایی هستند که اختلال را از دیدگاه فرد مبتلا بررسی می­کنند. پرسشنامه­ی­­ مقیاس فردی لکنت (sss) به غربالگری سه حیطه شدت لکنت، اجتناب و جایگاه کنترل در یک مجموعه­­ی واحد می­­پردازد که می­تواند ضرورت نیاز به ارزیابی بیشتر جنبه های مختلف لکنت را نشان دهد. هدف اين تحقيق، ترجمه و تعيين روايي و پايايي نسخه فارسي پرسشنامه­ی مقیاس فردی لکنت بود. روش بررسی: با اجراي يك مطالعه آزمايشي بر روي 17  فرد داراي لكنت و انجام يك مصاحبه نيمه ساختمند با آنها روايي صوري و ثبات دروني پرسشنامه­ی مقیاس فردی لکنت (Subjective stuttering scale:SSS) بررسي شد. درمرحله بعد20 فرد بزرگسال داراي لكنت پرسشنامه­ی تعديل يافته را به همراه آزمون فهرست ادراکی لکنت PSI)perception of stuttering inventory)،مقیاس جایگاه کنترل رفتار                         behavior scale LCB)locus of control) و محاسبه درصد لکنت از نمونه گفتاری ضبط شده­ی آنها به منظور بررسي روايي همزمان، تكميل نمودند. يافته ها: نتایج تحقیق نشان داد که با توجه به میزان  Cronbach's alpha محاسبه شده(α=0/92) مقیاس از پایایی بالایی برخوردار است. در بررسی روایی همزمان، بین مقیاس درصد هجاهای لکنت شده(SS%) و سوال 1 پرسشنامه « SSS» ( 67/0 r =01/0= α) همبستگی معنی­داری وجود دارد و همچنین بین زیرآزمون اجتناب در پرسشنامه « SSS» و زیرآزمون اجتناب در پرسشنامه  « PSI» همبستگی معنی­داری وجود دارد ( r = 0/75 و α = 0/05 )، اما  بین زیرآزمون حوزه کنترل پرسشنامه « SSS» و پرسشنامه «LCB» همبستگی معناداری وجود ندارد    r = 0/15). ). هم چنین مقیاس از روایی سازه­ی مناسبی برخوردار می باشد.   نتيجه گيري:  با توجه به نتایج بدست آمده مي­توان اظهار داشت كه نسخه فارسي مقیاس فردی لکنت از روایی و پایایی مناسب برخوردار است.

Title: شنوایی فضایی: ا لگوها و کارکردها

http://jrrs.mui.ac.ir/index.php/jrrs/article/view/1206

چکیده   مقدمه: شنوایی فضایی بخشی از قابلیت‌های دستگاه شنیداری مرکزی است که امکان جهت یابی و درک گفتار در نویز را به شنونده می دهد. شنوایی فضایی، توانایی توجه انتخابی بهاصوات رسیده از یک جهت و سرکوب همزمان صداهای رسیده از جهات دیگر را میسر می سازد. هدف اینبررسی، مرور بر مطالعاتی است که به مبانی، الگو‌سازی و کارکردهای شنوایی فضایی پرداخته اند.   مواد و روش ها: در این مطالعه مقالات مختلفپیرامون شنوایی فضایی بررسی شدند. از بانک‏های اطلاعاتی pubmed، Scopus، ProQuest، Google Scholar و واژه‏های کلیدی spatial hearingو spatial advantageو کلمات مرتبط با شنوایی فضایی در ترکیبات مختلف برای انجام جستجو استفاده گردید.   یافته ها: شنوایی فضایی به عنوان بک پدیده ی نوین پردازشی مطرح است و دارای کارکردهای مختلفی استو طی سالهای اخیر حجم انبوهی از اطلاعات در این زمینه فراهم شده است. این پدیدهفراورده ی پردازش دو گوشی است و کارکردهای آن در حیطه های مختلفی چون "درک گفتار در محیط شلوغ" ، پردازش مرکزی شنوایی، جهت یابی و درک فاصله به تدریج مورد تاکید قرار گرفته است.   نتیجه گیری: با توجه به اهمیت و کارکردهای شنوایی فضایی در پردازش شنوایی به خصوص در محیط های پیچیده ارزیابی، درمان و توانبخشی اختلالات شنوایی فضایی می تواند بخش مهمی از مشکلات افراد دارای اختلال پردازش شنوایی مرکزی را برطرف کند
